# Supplementary material for: Sex-Dependent Gut Microbiota Features and Functional Signatures in Metabolic Disfunction-Associated Steatotic Liver Disease
Source: Nutrients. 2024 Dec 4;16(23):4198. doi: 10.3390/nu16234198 (PMC11644196; doi:10.3390/nu16234198)
Supplement: Supplementary file 1 [file nutrients-16-04198-s001.zip › nutrients-3341341-supplementary.pdf]

nutrients

Supplementary Materials.

Sex-Dependent Gut Microbiota Features and Functional Signatures in Metabolic Disfunction-Associated Steatotic Liver Disease

Paola Mogna-Peláez [1], José I. Riezu-Boj [1,2], Fermin I. Milagro [1,2,3], Iñigo Clemente-Larramendi [1], Sergio Esteban Echeverría [1], José I. Herrero [2,4,5], Mariana Elorz [2,6], Alberto Benito-Boillos [2,6], Ana Luz Tobaruela-Resola [1], Pedro González-Muniesa [1,2,3], Josep A. Tur [3,7], J. Alfredo Martínez [3,8], Itziar Abete [1,2,3] and M. Angeles Zulet [1,2,3,*]

1. Department of Nutrition, Food Sciences and Physiology and Centre for Nutrition Research, Faculty of Pharmacy and Nutrition, University of Navarra, 31008 Pamplona, Spain; pmogna@unav.es (P.M.-P.); jiriezu@unav.es (J.I.R.-B.); fmilagro@unav.es (F.I.M.); iclemente.1@alumni.unav.es (I.C.-L.); sergioestebanecheverria@gmail.com (S.E.E.); atobaruela@unav.es (A.L.T.-R.); pgonmun@unav.es (P.G.-M.); iabetego@unav.es (I.A.)
2. Navarra Institute for Health Research (IdiSNA), 31008 Pamplona, Spain; iherrero@unav.es (J.I.H.); marelorz@unav.es (M.E.); albenitob@unav.es (A.B.-B.)
3. Biomedical Research Centre Network in Physiopathology of Obesity and Nutrition (CIBERobn), Instituto de Salud Carlos III, 28029 Madrid, Spain; pep.tur@uib.es (J.A.T.); jalfmtz@unav.es (J.A.M.)
4. Liver Unit, Clínica Universidad de Navarra, 31008 Pamplona, Spain
5. Biomedical Research Centre Network in Hepatic and Digestive Diseases (CIBERehd), 28029 Madrid, Spain
6. Department of Radiology, Clínica Universidad de Navarra, 31008 Pamplona, Spain
7. Research Group on Community Nutrition and Oxidative Stress, University of Balearic Islands-IUNICS & IDISBA, 07122 Palma, Spain
8. Precision Nutrition and Cardiovascular Health Program, IMDEA Food, CEI UAM + CSIC, 28049 Madrid, Spain

* Correspondence: mazulet@unav.es; Tel.: +34-948-425-600

Supplementary Table S1. Significant results from the MaAsLin2 differential abundance analysis in women.

WOMEN (n=68)					
Taxons		Increased in	Coefficient	P-value	Q-value
SPECIES	*Peptococcus simiae*	Controls	-1.086	1.96E-05	0.002
	Acholeplasma parvum	Controls	-1.016	1.31E-04	0.010
	Massiliprevotella massiliensis	Controls	-0.957	2.28E-03	0.044
	Parabacteroides gordonii	Controls	-0.714	7.72E-04	0.025
	Pseudoflavonifractor capillosus	Controls	-0.714	1.09E-03	0.027
	Intestinimonas massiliensis	Controls	-0.702	6.16E-04	0.023
	Harryflintia acetispora	Controls	-0.690	1.59E-05	0.002
	Sporobacter termitidis	Controls	-0.673	2.02E-04	0.012
	Anaerofilum pentosovorans	Controls	-0.605	1.49E-03	0.034
	Intestinimonas butyriciproducens	Controls	-0.595	9.01E-04	0.025
	Paludicola psychrotolerans	Controls	-0.465	2.18E-03	0.044
	Finegoldia magna	MASLD	0.540	2.32E-03	0.044
	Prevotella salivae	MASLD	0.667	2.78E-03	0.049
	Paramuribaculum intestinale	MASLD	0.722	9.27E-04	0.025
	Collinsella tanakaei	MASLD	0.862	2.76E-04	0.012
	Duncaniella muris	MASLD	1.019	2.37E-04	0.012
	Tyzzerella nexilis	MASLD	1.088	2.07E-05	0.002
GENUS	*Peptococcus*	Controls	-1.099	5.87E-06	0.001
	Massiliprevotella	Controls	-0.957	2.10E-03	0.031
	Pseudoflavonifractor	Controls	-0.714	1.06E-03	0.023
	Harryflintia	Controls	-0.691	8.30E-06	0.001
	Sporobacter	Controls	-0.673	1.43E-04	0.006
	Anaerofilum	Controls	-0.589	1.28E-03	0.025
	Intestinimonas	Controls	-0.559	2.85E-03	0.037
	Christensenella	Controls	-0.542	1.75E-04	0.006
	Paludicola	Controls	-0.465	1.41E-03	0.025
	Anaerotruncus	Controls	-0.384	3.22E-03	0.039
	Phocaeicola	MASLD	0.423	9.31E-04	0.023
	Sarcina	MASLD	0.482	2.25E-03	0.031
	Finegoldia	MASLD	0.540	1.92E-03	0.031
	Paramuribaculum	MASLD	0.721	9.02E-04	0.023
	Limosilactobacillus	MASLD	0.966	6.81E-05	0.003
	Tyzzerella	MASLD	1.088	1.91E-05	0.001
FAMILY	Desulfobacteraceae	Controls	-1.413	1.46E-06	<0.001
	Peptococcaceae 1	Controls	-1.102	4.98E-06	<0.001
	Christensenellaceae	Controls	-0.483	3.02E-04	0.006

Significantly different abundant taxa between MASLD and control groups. The increase in column refers to which group said taxa's abundance is incremented. The coefficient states the effect size of the analysis. The p-values from the analysis were corrected using FDR, as shown in the Q-value column.

Supplementary Table S2. Significant results from the MaAsLin2 differential abundance analysis in men.

MEN (n=66)					
Taxons		Increased in	Coefficient	P-value	Q-value
SPECIES	*Peptococcus simiae*	Controls	-1.086	1.02E-04	0.010
	Ruminococcus albus	Controls	-1.067	8.87E-05	0.010
	Ligilactobacillus ruminis	Controls	-0.936	2.08E-04	0.012
	Cloacibacillus porcorum	Controls	-0.806	1.80E-03	0.048
	Beduinibacterium massiliense	Controls	-0.720	5.14E-04	0.026
	Parabacteroides goldsteinii	Controls	-0.704	1.90E-03	0.048
	Parabacteroides gordonii	Controls	-0.686	6.63E-05	0.010
	Sporobacter termitidis	Controls	-0.684	8.07E-04	0.030
	Ruminococcus flavefaciens	Controls	-0.653	1.44E-03	0.048
	Harryflintia acetispora	Controls	-0.615	1.79E-04	0.012
	Ihubacter massiliensis	Controls	-0.380	1.91E-03	0.048
	Streptococcus gordonii	MASLD	0.832	7.66E-04	0.030
GENUS	*Peptococcus*	Controls	-1.193	4.21E-05	0.004
	Acholeplasma	Controls	-1.135	3.30E-05	0.004
	Ligilactobacillus	Controls	-0.789	1.77E-03	0.043
	Beduinibacterium	Controls	-0.720	3.83E-04	0.019
	Sporobacter	Controls	-0.685	5.97E-04	0.023
	Harryflintia	Controls	-0.616	1.34E-04	0.009
	Ihubacter	Controls	-0.380	1.63E-03	0.043
	Parabacteroides	MASLD	0.382	9.07E-04	0.029
FAMILY	Peptococcaceae 1	Controls	-1.194	2.77E-05	0.001
	Acholeplasmataceae	Controls	-1.133	3.02E-05	0.001
	Puniceicoccaceae	Controls	-0.756	2.46E-03	0.027
	Synergistaceae	Controls	-0.722	2.79E-03	0.027
	Oxalobacteraceae	Controls	-0.665	6.45E-03	0.042
	Porphyromonadaceae	MASLD	0.384	1.16E-03	0.017
	Streptococcaceae	MASLD	0.508	6.03E-03	0.042
	Anaeroplasmataceae	MASLD	0.748	4.65E-03	0.039

Significantly different abundant taxa between MASLD and control groups. The increase in column refers to which group said taxa's abundance is incremented. The coefficient states the effect size of the analysis. The p-values from the analysis were corrected using FDR, as shown in the Q-value column.

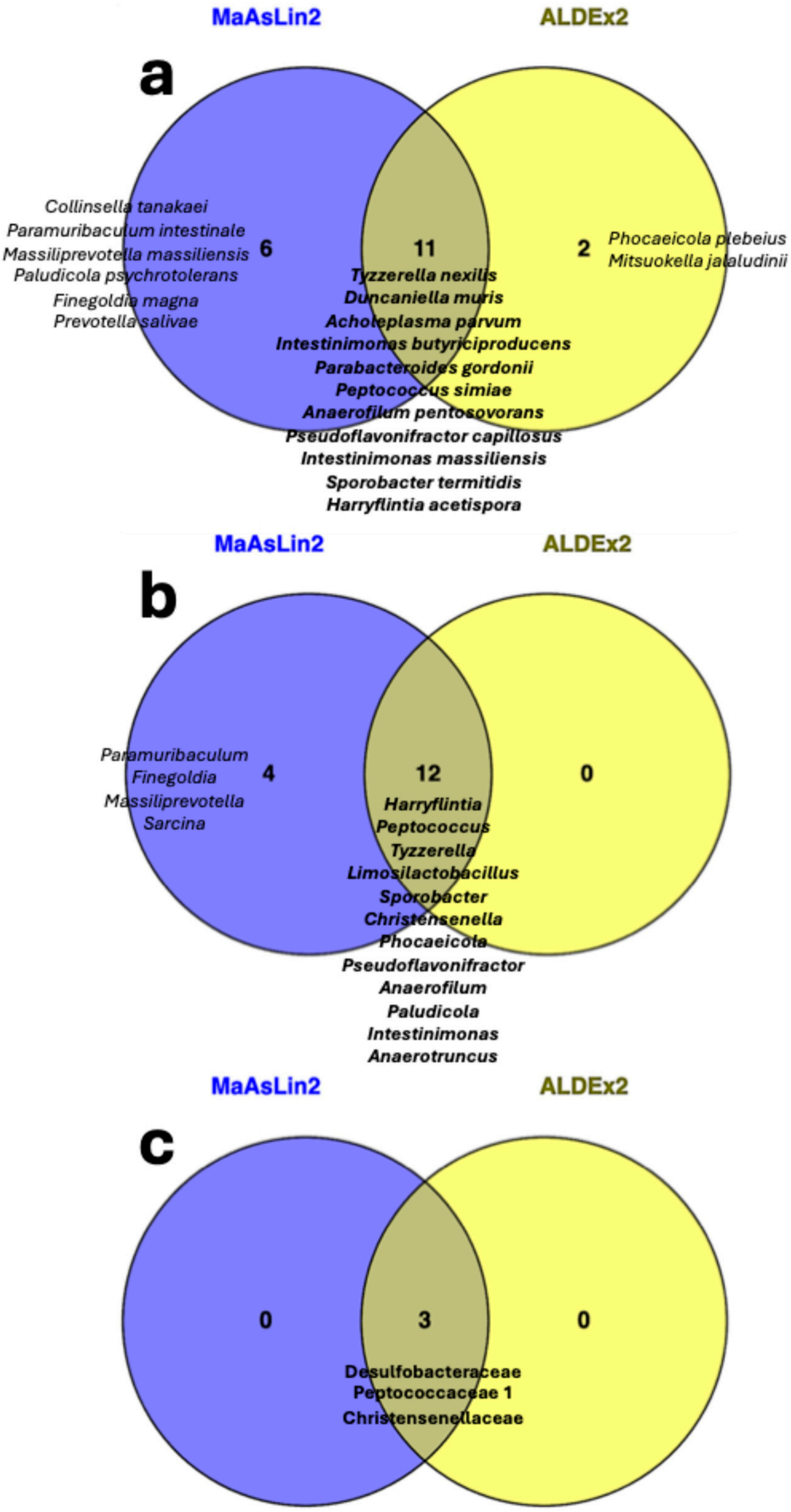

Supplementary Figure S1. Significant features obtained in differential abundance analyses in women: **a.** Significantly different species in ALDEx2 and MaAsLin2 analyses between control and MASLD women. **b.** Significantly different genera in ALDEx2 and MaAsLin2 analyses between control and MASLD women. **c.** Significantly different families in ALDEx2 and MaAsLin2 analyses between control and MASLD women.

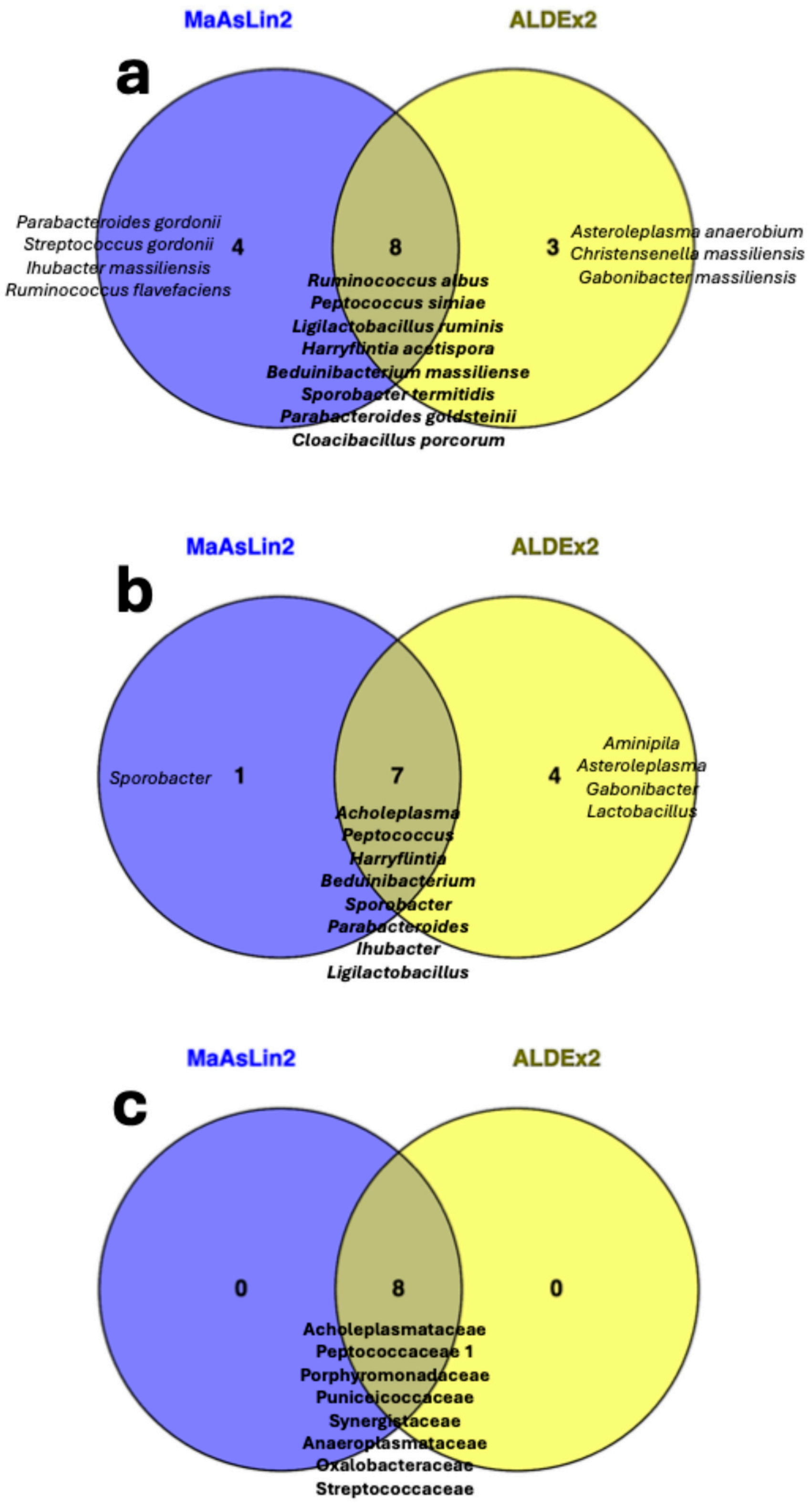

Supplementary Figure S2. Significant features obtained in differential abundance analyses in men: **a.** Significantly different species in ALDEx2 and MaAsLin2 analyses between control and MASLD men. **b.** Significantly different genera in ALDEx2 and MaAsLin2 analyses between control and MASLD men. **c.** Significantly different families in ALDEx2 and MaAsLin2 analyses between control and MASLD men.

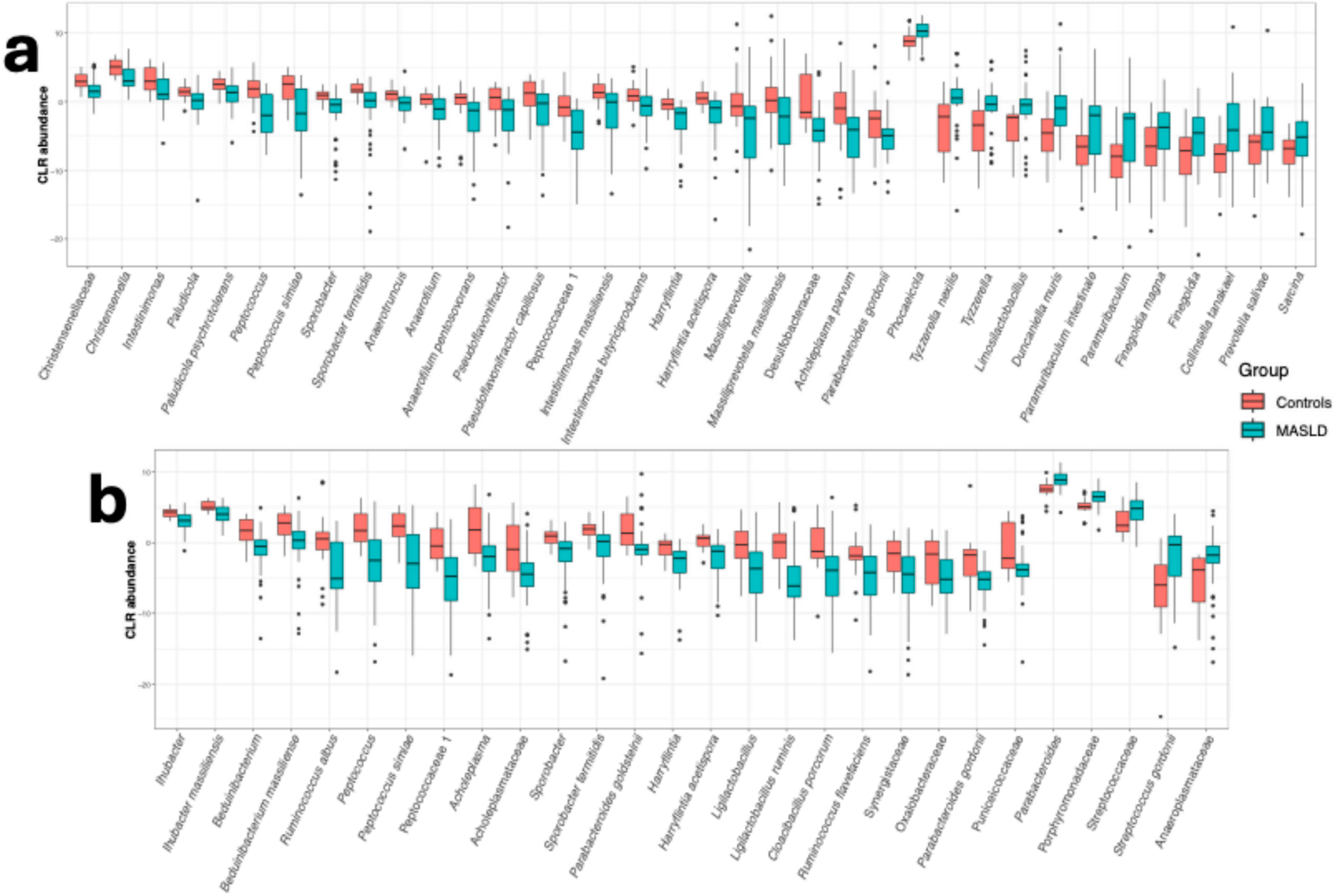

Supplementary Figure S3. CLR counts of MaAsLin2 differential abundance significant features in **a.** Women and **b.** Men.

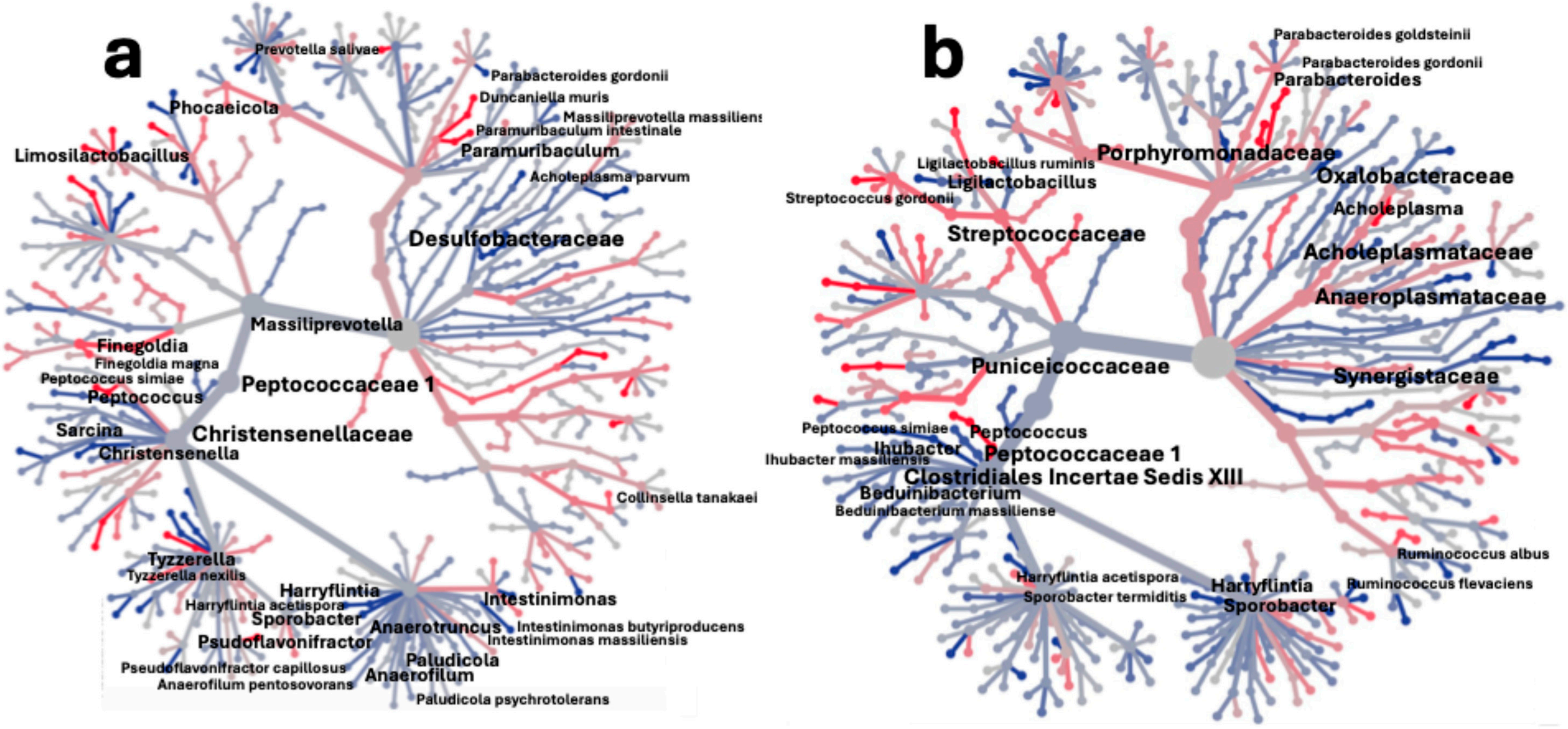

Supplementary Figure S4. Heat trees of MaAsLin2 differential abundance significant features in **a.** Women and **b.** Men

Supplementary Table S3. Q-values for the associations between metadata and significant microbial features in women.

	Tyzzerella nexilis	*Peptococcus simiae*	*Harryflintia acetispora*	*Acholeplasma parvum*	*Duncaniella muris*	*Collinsella tanakaei*	*Sporobacter termitidis*	*Intestinimonas massiliensis*	*Paramuribaculum intestinale*	*Intestinimonas*	*Parabacteroides gordonii*	*Pseudoflavonifractor capillosus*	*Anaerofilum pentosovorans*	*Massiliprevotella massiliensis*	*Paludicola psychrotolerans*	*Finegoldia magna*	*Prevotella salivae*	*Harryflintia*	*Peptococcus*	*Tyzzerella*	*Limosilactobacillus*	*Sporobacter*	*Christensenella*	*Paramuribaculum*	*Phocaeicola*	*Pseudoflavonifractor*	*Anaerofilum*	*Paludicola*	*Finegoldia*	*Massiliprevotella*	*Sarcina*	*Intestinimonas*	*Anaerotruncus*	*Desulfobacteraceae*	*Peptococcaceae 1*	*Christensenellaceae*
Weight	0.000	0.002	0.000	0.006	0.000	0.022	0.005	0.008	0.001	0.002	0.096	0.266	0.001	0.017	0.004	0.125	0.044	0.000	0.002	0.000	0.006	0.010	0.000	0.001	0.002	0.194	0.008	0.003	0.062	0.022	0.065	0.002	0.008	0.000	0.002	0.001
BMI	0.000	0.004	0.002	0.021	0.001	0.057	0.017	0.030	0.010	0.010	0.047	0.323	0.027	0.026	0.014	0.350	0.031	0.001	0.002	0.000	0.006	0.025	0.002	0.004	0.004	0.232	0.052	0.013	0.291	0.032	0.097	0.011	0.048	0.000	0.002	0.003
Waist	0.000	0.002	0.002	0.034	0.002	0.046	0.005	0.008	0.022	0.003	0.057	0.421	0.009	0.043	0.038	0.232	0.040	0.000	0.002	0.000	0.010	0.013	0.002	0.007	0.001	0.336	0.035	0.031	0.260	0.047	0.045	0.017	0.045	0.002	0.002	0.003
VAT	0.004	0.005	0.004	0.067	0.007	0.110	0.002	0.003	0.086	0.025	0.054	0.278	0.092	0.009	0.076	0.236	0.040	0.000	0.004	0.007	0.002	0.008	0.001	0.050	0.005	0.241	0.093	0.058	0.409	0.011	0.027	0.003	0.046	0.005	0.005	0.002
Fat mass (Kg)	0.000	0.002	0.001	0.028	0.000	0.020	0.005	0.013	0.003	0.011	0.064	0.257	0.006	0.029	0.008	0.150	0.125	0.000	0.002	0.000	0.011	0.012	0.000	0.002	0.002	0.168	0.017	0.008	0.101	0.040	0.048	0.002	0.027	0.000	0.001	0.001
FLI Index	0.000	0.006	0.010	0.020	0.012	0.097	0.014	0.005	0.044	0.002	0.063	0.520	0.014	0.017	0.049	0.294	0.033	0.002	0.002	0.000	0.006	0.030	0.001	0.012	0.002	0.394	0.048	0.034	0.388	0.019	0.067	0.023	0.022	0.014	0.004	0.002
Steatosis degree	0.001	0.000	0.005	0.005	0.009	0.030	0.002	0.012	0.115	0.005	0.048	0.024	0.014	0.021	0.027	0.048	0.054	0.001	0.000	0.002	0.000	0.005	0.001	0.066	0.011	0.020	0.013	0.013	0.109	0.017	0.059	0.016	0.005	0.000	0.000	0.002
Hepatic volume	0.021	0.028	0.031	0.010	0.020	0.705	0.031	0.001	0.112	0.001	0.293	0.239	0.033	0.037	0.123	0.148	0.169	0.012	0.024	0.024	0.009	0.071	0.002	0.144	0.001	0.242	0.101	0.101	0.242	0.028	0.053	0.002	0.007	0.120	0.020	0.004
Histological hepatic fat %	0.005	0.010	0.070	0.031	0.065	0.299	0.010	0.004	0.297	0.016	0.068	0.059	0.275	0.025	0.148	0.069	0.234	0.027	0.010	0.014	0.002	0.018	0.006	0.062	0.101	0.069	0.255	0.092	0.242	0.022	0.030	0.054	0.019	0.014	0.006	0.013
ALT	0.016	0.473	0.345	0.085	0.729	0.150	0.090	0.170	0.295	0.094	0.220	0.649	0.555	0.013	0.456	0.740	0.603	0.354	0.391	0.032	0.170	0.087	0.261	0.024	0.604	0.672	0.405	0.395	0.954	0.012	0.326	0.892	0.318	0.555	0.394	0.383
GGT	0.100	0.362	0.621	0.045	0.660	0.648	0.228	0.024	0.851	0.137	0.709	0.349	0.376	0.064	0.936	0.952	0.393	0.385	0.224	0.102	0.199	0.272	0.257	0.649	0.102	0.454	0.955	0.899	0.736	0.055	0.892	0.579	0.409	0.686	0.360	0.518
M30	0.096	0.176	0.393	0.332	0.363	0.031	0.062	0.795	0.153	0.863	0.768	0.553	0.361	0.633	0.524	0.090	0.053	0.378	0.251	0.094	0.200	0.039	0.324	0.438	0.717	0.479	0.695	0.481	0.232	0.677	0.070	0.500	0.554	0.036	0.183	0.182
CRP	0.002	0.011	0.024	0.194	0.013	0.121	0.164	0.085	0.102	0.002	0.123	0.097	0.003	0.043	0.068	0.200	0.073	0.004	0.007	0.005	0.018	0.141	0.002	0.051	0.034	0.047	0.008	0.031	0.040	0.039	0.241	0.044	0.048	0.001	0.007	0.003
Chemerin	0.211	0.025	0.214	0.438	0.641	0.772	0.154	0.123	0.772	0.367	0.632	0.582	0.317	0.553	0.275	0.514	0.919	0.114	0.043	0.329	0.225	0.230	0.032	0.367	0.193	0.497	0.291	0.226	0.971	0.393	0.235	0.204	0.441	0.817	0.061	0.090
LECT2	0.022	0.050	0.205	0.009	0.036	0.032	0.140	0.455	0.039	0.256	0.131	0.044	0.545	0.007	0.056	0.067	0.036	0.132	0.053	0.042	0.002	0.123	0.016	0.002	0.047	0.029	0.189	0.035	0.109	0.004	0.120	0.378	0.207	0.007	0.046	0.017
RBP4	0.301	0.140	0.276	0.733	0.951	0.303	0.090	0.108	0.360	0.385	0.130	0.163	0.588	0.301	0.189	0.006	0.853	0.165	0.101	0.289	0.632	0.079	0.056	0.027	0.446	0.107	0.410	0.143	0.185	0.398	0.586	0.127	0.098	0.500	0.097	0.036
Leptin	0.000	0.006	0.003	0.051	0.026	0.069	0.019	0.045	0.011	0.033	0.048	0.361	0.029	0.184	0.020	0.224	0.184	0.001	0.006	0.000	0.062	0.028	0.001	0.003	0.022	0.250	0.033	0.019	0.102	0.157	0.275	0.017	0.045	0.011	0.006	0.002
Adiponectin	0.000	0.004	0.013	0.137	0.019	0.125	0.009	0.004	0.096	0.003	0.052	0.137	0.012	0.011	0.033	0.313	0.427	0.003	0.010	0.002	0.004	0.017	0.008	0.089	0.077	0.126	0.002	0.030	0.579	0.011	0.129	0.005	0.002	0.000	0.008	0.010
Glucose	0.029	0.067	0.078	0.022	0.343	0.052	0.056	0.188	0.266	0.469	0.272	0.833	0.965	0.074	0.689	0.586	0.158	0.054	0.067	0.019	0.074	0.110	0.075	0.633	0.059	0.833	0.947	0.795	0.332	0.074	0.102	0.291	0.456	0.126	0.085	0.060
Insulin	0.007	0.017	0.025	0.088	0.191	0.060	0.027	0.010	0.230	0.022	0.021	0.229	0.320	0.028	0.042	0.048	0.291	0.011	0.013	0.010	0.024	0.043	0.002	0.139	0.032	0.218	0.207	0.031	0.061	0.017	0.561	0.023	0.115	0.018	0.017	0.003
HbA1c	0.120	0.255	0.128	0.003	0.323	0.086	0.291	0.405	0.582	0.456	1.000	0.991	0.369	0.014	0.809	0.441	0.154	0.124	0.200	0.132	0.154	0.390	0.183	0.362	0.253	0.904	0.916	0.745	0.313	0.012	0.187	0.883	0.218	0.388	0.129	0.173

HOMA Index	0.008	0.017	0.029	0.064	0.165	0.054	0.025	0.012	0.234	0.034	0.022	0.274	0.378	0.022	0.049	0.052	0.261	0.013	0.013	0.010	0.018	0.042	0.002	0.136	0.030	0.257	0.264	0.038	0.075	0.014	0.482	0.028	0.132	0.017	0.017	0.003
HDL Cholesterol	0.263	0.246	0.165	0.280	0.206	0.767	0.109	0.005	0.579	0.095	0.193	0.649	0.459	0.098	0.574	0.536	0.939	0.081	0.432	0.415	0.096	0.096	0.128	0.696	0.427	0.509	0.109	0.419	0.624	0.059	0.274	0.042	0.169	0.043	0.442	0.041
LDL Cholesterol	0.207	0.817	0.542	0.701	0.817	0.024	0.142	0.960	0.840	0.798	0.579	0.520	0.894	0.798	0.214	0.876	0.572	0.656	0.523	0.095	0.483	0.149	0.372	0.849	0.839	0.499	0.905	0.187	0.979	0.500	0.662	0.731	0.679	0.732	0.468	0.303
Tryglicerides	0.615	0.179	0.486	0.833	0.609	0.795	0.246	0.037	0.955	0.062	0.044	0.894	0.422	0.454	0.371	0.409	0.555	0.278	0.136	0.727	0.098	0.365	0.064	0.483	0.190	0.817	0.253	0.308	0.929	0.385	0.380	0.360	0.114	0.826	0.233	0.057

Data represents q-values (corrected p-values using FDR) of the correlations in Figure 3. a. Abbreviations: ALT. Alanine aminotransferase; ARFI. Acoustic Radiation Force elastography; AST. Aspartate aminotransferase; BMI. Body Mass Index; CRP. C-reactive protein; FLI. Fatty Liver Index; GGT. Gamma-glutamyl transferase; HbA1c; Glycated Hemoglobin; HDL. High-Density Lipoprotein; HOMA-IR. Homeostatic Model Assessment for Insulin Resistance; LECT2. Leukocyte cell-derived chemotaxin-2; LDL. Low-Density Lipoprotein; RBP4. Retinol Binding Protein 4; TE. transient elastography; VAT. Visceral Adipose Tissue. M30 and M65 are cytokeratin 18 (CK-18) antigens.

Supplementary Table S4. Q-values for the associations between metadata and significant microbial features in men.

	Ruminococcus albus	*Parabacteroides gordonii*	*Peptococcus simiae*	*Ligilactobacillus ruminis*	*Harryflintia acetispora*	*Beduinibacterium massiliense*	*Sporobacter termitidis*	*Parabacteroides goldsteinii*	*Ihubacter massiliensis*	*Ruminococcus flavefaciens*	*Cloacibacillus porcorum*	*Acholeplasma*	*Peptococcus*	*Harryflintia*	*Beduinibacterium*	*Sporobacter*	*Parabacteroides*	*Ihubacter*	*Ligilactobacillus*	*Acholeplasmataceae*	*Peptococcaceae 1*	*Porphyromonadaceae*	*Puniceicoccaceae*	*Synergistaceae*	*Anaeroplasmataceae*	*Oxalobacteraceae*
Weight	0.073	0.013	0.003	0.028	0.003	0.002	0.016	0.143	0.079	0.167	0.041	0.096	0.005	0.018	0.001	0.016	0.029	0.092	0.381	0.105	0.003	0.039	0.123	0.076	0.101	0.430
BMI	0.089	0.040	0.007	0.033	0.002	0.001	0.007	0.089	0.020	0.118	0.012	0.115	0.004	0.010	0.000	0.007	0.011	0.023	0.367	0.180	0.002	0.016	0.077	0.016	0.063	0.210
Waist	0.080	0.077	0.014	0.020	0.001	0.003	0.009	0.070	0.021	0.092	0.030	0.076	0.010	0.003	0.002	0.012	0.002	0.020	0.290	0.101	0.003	0.002	0.112	0.051	0.065	0.145
VAT	0.085	0.073	0.023	0.020	0.003	0.051	0.004	0.074	0.046	0.011	0.147	0.028	0.030	0.007	0.028	0.004	0.002	0.033	0.217	0.031	0.013	0.006	0.278	0.275	0.173	0.193
Fat mass (Kg)	0.134	0.106	0.003	0.007	0.000	0.002	0.003	0.117	0.010	0.050	0.011	0.096	0.002	0.003	0.001	0.005	0.001	0.012	0.156	0.110	0.001	0.001	0.122	0.032	0.145	0.286
FLI Index	0.112	0.109	0.003	0.036	0.003	0.000	0.003	0.109	0.003	0.143	0.033	0.058	0.007	0.012	0.000	0.004	0.003	0.003	0.492	0.096	0.003	0.004	0.067	0.057	0.076	0.244
Steatosis degree	0.010	0.126	0.011	0.019	0.006	0.047	0.004	0.118	0.028	0.027	0.120	0.047	0.015	0.008	0.034	0.003	0.023	0.019	0.123	0.066	0.003	0.044	0.168	0.143	0.022	0.081
Hepatic volume	0.176	0.091	0.041	0.142	0.003	0.057	0.012	0.150	0.077	0.187	0.298	0.025	0.080	0.005	0.045	0.008	0.430	0.056	0.579	0.060	0.017	0.447	0.611	0.180	0.072	0.090
Histological hepatic fat %	0.097	0.124	0.074	0.017	0.003	0.015	0.002	0.032	0.042	0.103	0.033	0.014	0.039	0.003	0.020	0.002	0.019	0.040	0.119	0.028	0.016	0.029	0.112	0.099	0.120	0.014
GGT	0.402	0.569	0.085	0.440	0.493	0.023	0.233	0.543	0.119	0.816	0.632	0.233	0.139	0.436	0.021	0.229	0.275	0.116	0.587	0.333	0.345	0.187	0.619	0.466	0.579	0.284
M30	0.181	0.044	0.084	0.152	0.142	0.116	0.045	0.088	0.587	0.229	0.313	0.370	0.051	0.121	0.081	0.039	0.198	0.546	0.081	0.349	0.059	0.277	0.022	0.798	0.233	0.011
M65	0.440	0.286	0.334	0.697	0.312	0.776	0.046	0.438	0.840	0.884	0.797	0.865	0.180	0.313	0.921	0.017	0.547	0.791	0.484	0.773	0.189	0.651	0.016	0.320	0.429	0.224
CRP	0.233	0.551	0.180	0.201	0.027	0.307	0.181	0.619	0.035	0.984	0.651	0.720	0.189	0.014	0.246	0.102	0.418	0.011	0.633	0.869	0.042	0.460	0.971	0.252	0.013	0.674
LECT2	0.062	0.023	0.013	0.003	0.019	0.009	0.060	0.136	0.044	0.096	0.135	0.011	0.025	0.026	0.004	0.047	0.001	0.032	0.021	0.031	0.002	0.001	0.584	0.055	0.220	0.665
RBP4	0.284	0.168	0.180	0.130	0.988	0.462	0.456	0.115	0.155	0.197	0.623	0.063	0.161	0.725	0.478	0.307	0.036	0.073	0.265	0.116	0.202	0.026	0.696	0.479	0.618	0.681
Leptin	0.538	0.418	0.016	0.003	0.007	0.019	0.036	0.145	0.061	0.115	0.014	0.219	0.003	0.019	0.005	0.042	0.001	0.065	0.116	0.252	0.003	0.000	0.122	0.098	0.104	0.431
Adiponectin	0.103	0.238	0.027	0.915	0.124	0.225	0.224	0.118	0.503	0.292	0.468	0.346	0.028	0.224	0.206	0.229	0.324	0.356	0.353	0.672	0.003	0.277	0.491	0.618	0.004	0.224
Insulin	0.042	0.146	0.065	0.013	0.080	0.122	0.048	0.356	0.086	0.129	0.108	0.129	0.056	0.062	0.050	0.041	0.089	0.057	0.078	0.097	0.033	0.072	0.214	0.147	0.081	0.137
HbA1c	0.003	0.538	0.137	0.463	0.095	0.204	0.070	0.180	0.096	0.125	0.467	0.067	0.045	0.093	0.159	0.064	0.028	0.083	0.466	0.040	0.071	0.040	0.233	0.455	0.123	0.329
HOMA Index	0.043	0.181	0.091	0.022	0.081	0.153	0.054	0.495	0.073	0.072	0.275	0.088	0.074	0.065	0.074	0.042	0.072	0.040	0.075	0.078	0.053	0.064	0.366	0.187	0.061	0.286
Total cholesterol	0.482	0.538	0.264	0.028	0.067	0.694	0.229	0.966	0.131	0.284	0.792	0.601	0.204	0.068	0.718	0.187	0.436	0.044	0.019	0.421	0.127	0.456	0.898	0.331	0.568	0.132
LDL Cholesterol	0.154	0.799	0.224	0.028	0.150	0.843	0.150	0.543	0.064	0.111	0.458	0.497	0.168	0.211	0.741	0.125	0.206	0.025	0.021	0.358	0.134	0.197	0.839	0.224	0.998	0.252
Tryglicerides	0.394	0.670	0.051	0.226	0.130	0.017	0.040	0.619	0.006	0.295	0.508	0.122	0.228	0.170	0.011	0.036	0.090	0.010	0.992	0.266	0.126	0.096	0.430	0.402	0.468	0.735

Data represents q-values (corrected p-values using FDR) of the correlations in Figure 3. b. Abbreviations: ALT. Alanine aminotransferase; ARFI. Acoustic Radiation Force elastography; AST. Aspartate aminotransferase; BMI. Body Mass Index; CRP. C-reactive protein; FLI. Fatty Liver Index; GGT. Gamma-glutamyl

transferase; HbA1c; Glycated Hemoglobin; HDL. High-Density Lipoprotein; HOMA-IR. Homeostatic Model Assessment for Insulin Resistance; LECT2. Leukocyte cell-derived chemotaxin-2; LDL. Low-Density Lipoprotein; RBP4. Retinol Binding Protein 4; TE. transient elastography; VAT. Visceral Adipose Tissue. M30 and M65 are cytokeratin 18 (CK-18) antigens.

Supplementary Table S5. Significant results for the predicted functional analysis in women.

WOMEN (n=68)						
Main function	Feature	Description	Increased in	Coef	p-value	q-value
Aldehyde Degradation	METHGLYUT-PWY	Superpathway of methylglyoxal degradation	MASLD	0.986	1.83E-03	3.26E-02
Amines and Polyamines Biosynthesis	PWY-6562*	Norspermidine biosynthesis	MASLD	2.485	3.23E-05	1.04E-03
	ORNDEG-PWY	Superpathway of ornithine degradation	MASLD	1.753	1.43E-06	1.70E-04
Amino Acid Biosynthesis	GLUTORN-PWY	L-ornithine biosynthesis	Controls	-0.077	3.94E-03	4.96E-02
	ARGSYNBSUB-PWY	L-arginine biosynthesis II (acetyl cycle)	Controls	-0.076	4.02E-03	4.96E-02
	DAPLYSINESYN-PWY*	L-lysine biosynthesis I	Controls	-0.073	3.04E-03	4.70E-02
	PWY-6629	Superpathway of L-tryptophan biosynthesis	MASLD	1.138	3.53E-04	7.84E-03
Amino Acid Degradation	ARGDEG-PWY	Superpathway of L-arginine. putrescine. and 4-aminobutanoate degradation	MASLD	1.590	7.39E-06	4.39E-04
	ORNARGDEG-PWY	Superpathway of L-arginine and L-ornithine degradation	MASLD	1.590	7.39E-06	4.39E-04
	AST-PWY	L-arginine degradation II (AST pathway)	MASLD	1.642	1.05E-05	4.66E-04
	THREOCAT-PWY*	Superpathway of L-threonine metabolism	MASLD	1.878	9.04E-06	4.60E-04
Aromatic Compounds Degradation	PWY-5415	Catechol degradation I (meta-cleavage pathway)	MASLD	0.816	1.24E-03	2.32E-02
	PWY-6182*	Superpathway of salicylate degradation	MASLD	1.740	3.98E-03	4.96E-02
	PWY-6185*	4-methylcatechol degradation (ortho cleavage)	MASLD	1.826	4.04E-03	4.96E-02
	PWY0-321*	Phenylacetate degradation I (aerobic)	MASLD	2.457	4.79E-05	1.35E-03
	3-HYDROXYPHENYLACETATE-DEGRADATION-PWY*	4-hydroxyphenylacetate degradation	MASLD	2.461	1.31E-05	5.18E-04
	PWY-6071*	Superpathway of phenylethylamine degradation	MASLD	2.472	4.95E-05	1.35E-03
Cell Structures Biosynthesis	ECASYN-PWY	Enterobacterial common antigen biosynthesis	MASLD	1.587	6.87E-06	4.39E-04
Fatty Acid and Lipid Biosynthesis	LPSSYN-PWY	Superpathway of lipopolysaccharide biosynthesis	MASLD	1.083	9.37E-05	2.38E-03
Fermentation	PWY-6588*	Pyruvate fermentation to acetone	Controls	-0.297	3.31E-03	4.91E-02
	PWY-5676	Acetyl-CoA fermentation to butanoate II	Controls	-0.214	6.36E-04	1.33E-02
Nucleotide Degradation	PWY-6353*	Purine nucleotides degradation II (aerobic)	Controls	-0.146	3.53E-03	4.96E-02
Quinol and Quinone Biosynthesis	PWY-7373	Superpathway of demethylmenaquinol-6 biosynthesis II	MASLD	2.160	1.15E-03	2.27E-02
Siderophore Biosynthesis	ENTBACSYN-PWY	Enterobactin biosynthesis	MASLD	1.382	1.66E-05	5.91E-04
	AEROBACTINSYN-PWY*	Aerobactin biosynthesis	MASLD	3.963	9.88E-08	2.46E-05
Sugar Derivatives Degradation	GALACTARDEG-PWY	D-galactarate degradation I	MASLD	0.639	2.29E-03	3.71E-02
	GLUCARGALACTSUPER-PWY	Superpathway of D-glucarate and D-galactarate degradation	MASLD	0.639	2.29E-03	3.71E-02
	PWY-7446	Sulfoglycolysis	MASLD	0.639	2.29E-03	3.71E-02
	KETOGLUCONMET-PWY	Ketogluconate metabolism	MASLD	2.155	1.38E-07	2.46E-05

Data from Picrust2 predicted pathways comparative analysis of controls vs. MASLD group. Features marked with an * were commonly found in the significant results in both sexes. The increase in column refers to which group said taxa's abundance is incremented. The coefficient states the effect size of the analysis. The p-values from the analysis were corrected using FDR, as shown in the Q-value column.

Supplementary Table S6. Significant results for the predicted functional analysis in men.

MEN (n=66)						
Main function	Feature	Description	Increased in	Coef	p-value	q-value
Methanogenesis	METH-ACETATE-PWY	Methanogenesis from acetate	Controls	-0.373	2.35E-03	2.81E-02
Amines and Polyamines Biosynthesis	PWY-6562*	Norspermidine biosynthesis	MASLD	2.818	6.32E-06	3.23E-04
Amino Acid Biosynthesis	DAPLYSINESYN-PWY*	L-lysine biosynthesis I	Controls	-0.129	6.53E-04	1.38E-02
	COMPLETE-ARO-PWY	Superpathway of aromatic amino acid biosynthesis	Controls	-0.068	4.04E-03	4.14E-02
	PWY-5154	L-arginine biosynthesis III (via N-acetyl-L-citrulline)	MASLD	0.222	3.35E-03	3.75E-02
Amino Acid Degradation	THREOCAT-PWY*	Superpathway of L-threonine metabolism	MASLD	2.292	1.00E-06	8.99E-05
Aromatic Compounds Biosynthesis	ARO-PWY	Chorismate biosynthesis I	Controls	-0.068	3.78E-03	4.10E-02
Aromatic Compounds Degradation	PROTOCATECHUATE-ORTHO-CLEAVAGE-PWY	Protocatechuate degradation II (ortho-cleavage pathway)	MASLD	1.767	2.71E-04	6.48E-03
	CATECHOL-ORTHO-CLEAVAGE-PWY	Catechol degradation	MASLD	2.370	2.98E-04	6.67E-03
	PWY-5417	Catechol degradation III (ortho-cleavage pathway)	MASLD	2.435	2.09E-04	5.76E-03
	PWY-5431	Aromatic compounds degradation via 3-oxodipate	MASLD	2.435	2.09E-04	5.76E-03
	3-HYDROXYPHENYLACETATE-DEGRADA-TION-PWY*	4-hydroxyphenylacetate degradation	MASLD	2.467	7.57E-05	2.71E-03
	PWY-6182*	Superpathway of salicylate degradation	MASLD	2.519	1.01E-04	3.28E-03
	PWY0-321*	Phenylacetate degradation I (aerobic)	MASLD	2.678	2.64E-05	1.05E-03
	PWY-6071*	Superpathway of phenylethylamine degradation	MASLD	2.769	1.65E-05	7.40E-04
	PWY-5181	Toluene degradation III (aerobic) (via p-cresol)	MASLD	3.007	4.83E-06	2.88E-04
	PWY-6185*	4-methylcatechol degradation (ortho cleavage)	MASLD	3.079	1.85E-06	1.33E-04
	PWY-5178	Toluene degradation IV (aerobic) (via catechol)	MASLD	3.344	2.36E-04	6.04E-03
	GALLATE-DEGRADATION-II-PWY	Gallate degradation I	MASLD	4.979	6.04E-08	7.21E-06
	METHYLGALLATE-DEGRADATION-PWY	Methylgallate degradation	MASLD	4.984	3.07E-09	5.49E-07
	GALLATE-DEGRADATION-I-PWY	Gallate degradation II	MASLD	4.985	2.39E-09	5.49E-07
Cell Structures Biosynthesis	PWY0-1586	Peptidoglycan maturation	Controls	-0.196	5.49E-03	4.92E-02
Fatty Acid and Lipid Biosynthesis	PWY4FS-7	Phosphatidylglycerol biosynthesis I (plastidic)	Controls	-0.114	1.83E-03	2.62E-02
	PWY4FS-8	Phosphatidylglycerol biosynthesis II (non-plastidic)	Controls	-0.114	1.83E-03	2.62E-02
	PHOSLIPSYN-PWY	Superpathway of phospholipid biosynthesis I (bacteria)	Controls	-0.089	1.48E-03	2.31E-02
	PWY0-1319	CDP-diacylglycerol biosynthesis II	Controls	-0.079	1.31E-03	2.13E-02
	PWY-5667	CDP-diacylglycerol biosynthesis I	Controls	-0.079	1.31E-03	2.13E-02
Fermentation	PWY-6588*	Pyruvate fermentation to acetone	Controls	-0.364	2.04E-03	2.81E-02
	PWY-5100	Pyruvate fermentation to acetate and lactate II	Controls	-0.091	2.16E-03	2.81E-02
Energy Generation	PWY-5741	Ethylmalonyl-CoA pathway	MASLD	1.313	4.46E-03	4.20E-02
Nucleotide Degradation	SALVADEHYPOX-PWY	Adenosine nucleotides degradation II	Controls	-0.270	4.27E-03	4.14E-02
	P164-PWY	Purine nucleobases degradation I (anaerobic)	Controls	-0.208	2.35E-03	2.81E-02
	PWY-6353*	Purine nucleotides degradation II (aerobic)	Controls	-0.201	4.28E-03	4.14E-02
	PWY0-1297	Superpathway of purine deoxyribonucleosides degradation	Controls	-0.157	5.68E-03	4.96E-02

	PWY0-1298	Superpathway of pyrimidine deoxyribonucleosides degradation	Controls	-0.153	5.32E-03	4.88E-02
Porphyrin Compounds Biosynthesis	HEMESYN2-PWY	Heme biosynthesis II (anaerobic)	MASLD	0.427	1.19E-03	2.13E-02
Siderophore Biosynthesis	AEROBACTINSYN-PWY*	Aerobactin biosynthesis	MASLD	2.510	8.26E-04	1.56E-02
Terpenoids Biosynthesis	PWY-7391	Isoprene biosynthesis II	Controls	-1.221	6.96E-04	1.38E-02
Tetrapyrrole Biosynthesis	PWY-5188	Tetrapyrrole biosynthesis I (from glutamate)	Controls	-0.186	2.33E-03	2.81E-02
Vitamins Biosynthesis	BIOTIN-BIOSYNTHESIS-PWY	Biotin biosynthesis I	MASLD	0.293	3.10E-03	3.58E-02
	PWY-6519	8-amino-7-oxononanoate biosynthesis I	MASLD	0.313	4.01E-03	4.14E-02

Data from Picrust2 predicted pathways comparative analysis of controls vs. MASLD group. Features marked with an * were commonly found in the significant results in both sexes. The increase in column refers to which group said taxa's abundance is incremented. The coefficient states the effect size of the analysis. The p-values from the analysis were corrected using FDR, as shown in the Q-value column.

Supplementary Table S7. Q-values for the associations between altered metabolic pathways and significant microbial features in women.

	Tyzzerella nexilis	Peptococcus simiae	Harryflintia acetispora	Sporobacter termitidis	Intestinimonas massiliensis	Intestinimonas butyriciproducens	Pseudoflavonifractor capillosus	Anaerofilum pentosovorans	Massiliprevotella massiliensis	Paludicola psychrotolerans	Finegoldia magna	Harryflintia	Peptococcus	Sporobacter	Christensenella	Phocaeicola	Pseudoflavonifractor	Anaerofilum	Paludicola	Massiliprevotella	Intestinimonas	Anaerotruncus	Peptococcaceae 1	Christensenellaceae
Superpathway of ornithine degradation	0.178	0.745	0.633	0.938	0.856	0.684	0.958	0.809	0.168	0.928	0.023	0.998	0.706	0.879	0.510	0.543	0.770	0.627	0.987	0.099	0.823	0.859	0.822	0.559
Enterobacterial common antigen biosynthesis	0.142	0.689	0.649	0.985	0.895	0.617	0.996	0.832	0.141	0.983	0.022	0.998	0.680	0.843	0.395	0.606	0.848	0.626	0.949	0.084	0.725	0.907	0.805	0.461
Superpathway of L-arginine. putrescine. and 4-aminobutanoate degradation	0.130	0.869	0.564	0.869	0.840	0.682	0.933	0.655	0.209	0.872	0.020	0.951	0.895	0.928	0.586	0.577	0.907	0.771	0.936	0.138	0.706	0.953	0.979	0.661
Superpathway of L-arginine and L-ornithine degradation	0.130	0.869	0.564	0.869	0.840	0.682	0.933	0.655	0.209	0.872	0.020	0.951	0.895	0.928	0.586	0.577	0.907	0.771	0.936	0.138	0.706	0.953	0.979	0.661
L-arginine degradation II	0.118	0.706	0.626	0.985	0.880	0.645	0.998	0.878	0.137	0.944	0.025	0.996	0.698	0.842	0.440	0.614	0.843	0.568	0.981	0.082	0.734	0.896	0.828	0.525
Superpathway of lipopolysaccharide biosynthesis	0.059	0.264	0.898	0.932	0.869	0.582	0.317	0.866	0.041	0.802	0.066	0.891	0.340	0.845	0.176	0.840	0.221	0.729	0.787	0.032	0.938	0.859	0.362	0.304
Sulfoglycolysis	0.075	0.706	0.559	0.920	0.981	0.548	0.933	0.865	0.047	0.915	0.015	0.932	0.765	0.952	0.419	0.652	0.764	0.715	0.986	0.031	0.596	0.910	0.841	0.554
Superpathway of L-tryptophan biosynthesis	0.069	0.652	0.673	0.946	0.847	0.462	0.843	0.765	0.099	0.989	0.013	0.965	0.704	0.985	0.321	0.629	0.655	0.809	0.974	0.081	0.493	0.761	0.736	0.411
Acetyl-CoA fermentation to butanoate II	0.357	0.001	0.006	0.000	0.012	0.024	0.015	0.050	0.062	0.000	0.649	0.003	0.001	0.000	0.000	0.013	0.039	0.022	0.000	0.134	0.024	0.014	0.002	0.000
Catechol degradation I	0.037	0.521	0.981	0.781	0.886	0.444	0.991	0.844	0.031	0.596	0.293	0.577	0.706	0.567	0.105	0.951	0.844	0.329	0.519	0.019	0.176	0.673	0.840	0.262
Superpathway of methylglyoxal degradation	0.092	0.842	0.606	0.843	0.910	0.629	0.946	0.715	0.109	0.840	0.028	0.974	0.931	0.989	0.517	0.729	0.915	0.947	0.855	0.082	0.557	0.989	0.985	0.682
L-lysine biosynthesis I	0.582	0.024	0.260	0.004	0.221	0.586	0.225	0.366	0.369	0.035	0.876	0.185	0.041	0.014	0.074	0.296	0.329	0.052	0.031	0.521	0.706	0.265	0.022	0.050
Pyruvate fermentation to acetone	0.414	0.006	0.000	0.000	0.001	0.011	0.084	0.047	0.084	0.000	0.698	0.000	0.005	0.000	0.000	0.001	0.165	0.013	0.000	0.170	0.001	0.002	0.012	0.000
Purine nucleotides degradation II	0.736	0.014	0.026	0.000	0.018	0.042	0.057	0.105	0.570	0.002	0.843	0.012	0.007	0.002	0.012	0.138	0.118	0.008	0.002	0.805	0.226	0.012	0.006	0.004
L-ornithine biosynthesis	0.559	0.041	0.220	0.003	0.173	0.681	0.255	0.436	0.349	0.041	0.933	0.140	0.077	0.012	0.078	0.279	0.357	0.066	0.038	0.495	0.596	0.220	0.054	0.060
L-arginine biosynthesis II	0.708	0.034	0.240	0.002	0.220	0.770	0.219	0.405	0.302	0.040	0.926	0.180	0.068	0.010	0.049	0.129	0.310	0.059	0.041	0.444	0.677	0.376	0.053	0.041

The data represent q-values (corrected p-values using FDR) of the correlations described in Figure 4. a.

Supplementary Table S8. Q-values for the associations between altered metabolic pathways and significant microbial features in men.

	Ruminococcus albus	*Peptococcus simiae*	*Ligilactobacillus ruminis*	*Harryflintia acetispora*	*Beduinibacterium massiliense*	*Streptococcus gordonii*	*Sporobacter termitidis*	*Parabacteroides goldsteinii*	*Ihubacter massiliensis*	*Cloacibacillus porcorum*	*Acholeplasma*	*Peptococcus*	*Harryflintia*	*Beduinibacterium*	*Sporobacter*	*Parabacteroides*	*Ihubacter*	*Ligilactobacillus*	*Acholeplasmataceae*	*Peptococcaceae 1*	*Porphyromonadaceae*	*Puniceicoccaceae*	*Synergistaceae*	*Oxalobacteraceae*	*Streptococcaceae*
Gallate degradation I	0.723	0.522	0.449	0.576	0.175	0.132	0.899	0.243	0.634	0.075	0.966	0.402	0.532	0.041	0.626	0.797	0.400	0.898	0.996	0.452	0.825	0.585	0.224	0.306	0.898
Methylgallate degradation	0.723	0.522	0.449	0.576	0.175	0.132	0.899	0.243	0.634	0.075	0.966	0.402	0.532	0.041	0.626	0.797	0.400	0.898	0.996	0.452	0.825	0.585	0.224	0.306	0.898
Superpathway of L-threonine metabolism	0.860	0.683	0.573	0.615	0.949	0.243	0.412	0.196	0.208	0.252	0.804	0.709	0.305	0.699	0.175	0.966	0.037	0.398	0.421	0.638	0.778	0.397	0.461	0.135	0.021
Norspermidine biosynthesis	0.961	0.941	0.333	0.839	0.977	0.402	0.939	0.429	0.226	0.517	0.948	0.989	0.535	0.651	0.584	0.965	0.025	0.758	0.837	0.988	0.825	0.599	0.630	0.179	0.514
Toluene degradation IV	0.881	0.630	0.991	0.865	0.988	0.012	0.898	0.593	0.961	0.426	0.976	0.693	0.989	0.824	0.535	0.368	0.637	0.762	0.772	0.585	0.158	0.544	0.998	0.858	0.844
Protocatechuate degradation II	0.857	0.637	0.650	0.477	0.563	0.704	0.767	0.605	0.989	0.443	0.393	0.443	0.556	0.811	0.971	0.548	0.742	0.612	0.511	0.693	0.273	0.844	0.598	0.030	0.720
Isoprene biosynthesis II	0.555	0.371	0.599	0.351	0.001	0.868	0.175	0.915	0.693	0.040	0.428	0.166	0.444	0.001	0.171	0.013	0.852	0.857	0.482	0.226	0.008	0.002	0.010	0.778	0.009
Aerobactin biosynthesis	0.881	0.975	0.386	0.998	0.779	0.700	0.959	0.711	0.257	0.695	0.722	0.960	0.799	0.966	0.755	0.597	0.028	0.693	0.941	0.997	0.615	0.939	0.694	0.113	0.287
Heme biosynthesis II	0.939	0.708	0.522	0.080	0.022	0.008	0.038	0.186	0.016	0.683	0.001	0.852	0.073	0.045	0.046	0.161	0.022	0.728	0.000	0.670	0.433	0.495	0.146	0.696	0.619
Pyruvate fermentation to acetone	0.912	0.009	0.045	0.017	0.001	0.755	0.003	0.780	0.000	0.019	0.005	0.002	0.055	0.005	0.006	0.062	0.001	0.058	0.080	0.000	0.121	0.053	0.009	0.264	0.010
Purine nucleobases degradation I	0.844	0.085	0.044	0.615	0.730	0.711	0.257	0.961	0.195	0.226	0.475	0.028	0.832	0.786	0.307	0.204	0.253	0.022	0.502	0.049	0.150	0.182	0.097	0.981	0.202
Biotin biosynthesis I	0.015	0.511	0.659	0.003	0.008	0.180	0.000	0.105	0.000	0.738	0.022	0.472	0.002	0.015	0.001	0.000	0.000	0.868	0.040	0.113	0.001	0.211	0.847	0.803	0.475
L-arginine biosynthesis III	0.472	0.814	0.429	0.040	0.034	0.116	0.003	0.034	0.002	0.676	0.007	0.707	0.031	0.066	0.008	0.000	0.003	0.853	0.006	0.506	0.000	0.419	0.672	0.693	0.584
8-amino-7-oxononanoate biosynthesis I	0.011	0.574	0.718	0.002	0.008	0.223	0.000	0.132	0.000	0.768	0.036	0.556	0.002	0.014	0.000	0.000	0.000	0.761	0.077	0.132	0.002	0.200	0.919	0.811	0.549
Adenosine nucleotides degradation II	0.989	0.062	0.005	0.258	0.035	0.722	0.266	0.693	0.150	0.133	0.040	0.014	0.555	0.069	0.406	0.164	0.211	0.007	0.147	0.013	0.194	0.186	0.031	0.966	0.141
Purine nucleotides degradation II	0.884	0.146	0.017	0.389	0.188	0.989	0.336	0.837	0.227	0.186	0.102	0.046	0.672	0.249	0.442	0.327	0.332	0.013	0.259	0.050	0.388	0.185	0.075	0.977	0.237
Peptidoglycan maturation	0.329	0.717	0.012	0.968	0.755	0.755	0.961	0.778	0.502	0.529	0.976	0.406	0.859	0.693	0.941	0.697	0.598	0.118	0.798	0.477	0.655	0.219	0.220	0.837	0.690

Data represents q-values (corrected p-values using FDR) of the correlations in Figure 4. b.
